# Supplementary material for: The Relationship between Nkx2.1 and DNA Oxidative Damage Repair in Nickel Smelting Workers: Jinchang Cohort Study
Source: Int J Environ Res Public Health. 2019 Jan 4;16(1):120. doi: 10.3390/ijerph16010120 (PMC6339211; doi:10.3390/ijerph16010120)
Supplement: Supplementary file 1 [file ijerph-16-00120-s001.pdf]

Supplement Table 1. The age and service length distribution of all 969 nickel smelters

Service length	Ages							Total
	20~	25~	30~	35~	40~	45~	50~	
0~	**20**	4	12	19	28	22	4	109
5~	0	**20**	0	21	21	30	9	101
10~	0	1	**20**	50	17	21	6	115
15~	0	0	5	**80**	57	42	11	195
20~	0	0	1	36	**72**	26	4	139
25~	0	0	0	1	50	**192**	28	271
30~	0	0	0	0	1	17	**21**	39
Total	20	25	38	207	246	350	83	969

Supplement Table 2. The age and service length distribution of 140 sampling nickel smelters

Service length	Ages							Total
	20~	25~	30~	35~	40~	45~	50~	
0~	**20**	—	—	—	—	—	—	20
5~	—	**20**	—	—	—	—	—	20
10~	—	—	**20**	—	—	—	—	20
15~	—	—	—	**20**	—	—	—	20
20~	—	—	—	—	**20**	—	—	20
25~	—	—	—	—	—	**20**	—	20
30~	—	—	—	—	—	—	**20**	20
Total	20	20	20	20	20	20	20	140

Supplement Table 3. Expression of 8-OHdG and hOGG1 in difference service length categories between nickel smelters group and administrative officers group.

Service length(years)	8-OHdG(ng/ml)				hOGG1(U/ml)			
	Nickel smelters		Administrative officers		Nickel smelters		Administrative officers	
	n	x±st.d	n	x±st.d	n	x±st.d	n	x±st.d
**~4**	20	0.1785±0.0119	20	0.1474±0.0231	20	0.0568±0.0451	20	0.0912±0.0470
**5~9**	20	0.1686±0.0119	20	0.1439±0.0291	20	0.0593±0.0109	20	0.0961±0.0694
**10~14**	20	0.1838±0.0124	20	0.1437±0.0230	20	0.0765±0.0607	20	0.0974±0.0724
**15~19**	20	0.1653±0.0118	20	0.1406±0.0270	20	0.0584±0.0089	20	0.1024±0.0766
**20~24**	20	0.1631±0.0168	20	0.1449±0.0213	20	0.0482±0.0197	20	0.0813±0.0206
**25~29**	20	0.1552±0.0166	20	0.1471±0.0203	20	0.0517±0.0052	20	0.0824±0.0290
**30~**	20	0.1628±0.0180	20	0.1472±0.0189	20	0.0464±0.0095	20	0.0939±0.0273
**Total**	140	0.1682±0.0143	140	0.145±0.0247	140	0.0567±0.0311	140	0.0921±0.0530
***Z* (Wilcoxon)**	8.688				-8.947			
p	<0.001				<0.001			
***F* (ANOVA)**	3.524		0.6		2.194		0.425	
p	0.003		0.730		0.047		0.861	
**r_s (Spearman)**	-0.239		-0.026		-0.064		0.137	
p	0.005		0.759		0.453		0.107	

Note: **x* represents the mean of 8-OHdG concentration. *St.d* represents the standard deviation
